# Bright’s Disease of the Kidneys

**Published:** 1846-09

**Authors:** Geo. N. Burwell


### BUFFALO MEDICAL JOURNAL

AND

### MONTHLY REVIEW.

**VOL. 2.** SEPTEMBER, 1846. **NO. 4,**

### ORIGINAL COMMUNICATIONS.

ART. I.—*Bright's Disease of the Kidneys.* By Geo. N. Burwell, M. D. (*Continued from page* 132.)

*Symptoms.*—In common with most other diseases, Bright's disease presents itself in two forms, the acute and the chronic. The acute form is most frequently met with as a sequela of scarlet fever, and is, therefore, more common in children than in adults. In the latter it is generally known under the name of "Acute or febrile Dropsy." It is generally preceded for a day or two by general uneasiness, anorexia, and headache; the face becomes swollen, the pulse rises, and soon decided febrile symptoms set in, preceded frequently by a chill, more or less severe. The chill, however, does not appear to be a common symptom in cases succeeding Scarlet Fever.

Occasionally the attack is sudden and violent—high febrile symptoms, a rapid effusion into the cellular tissue, great deficiency of urinary secretion, coma and convulsions follow each other in rapid succession. All such cases partake of an inflammatory nature in a marked degree, and unless the antiphlogistic treatment is adopted, promptly and decidedly, in proportion to the severity of the attack, convulsions, persistent coma, or pleurisy with hydrothorax, or pneumonia, or peritonitis, will be apt soon to complicate the dropsical effusion, and prove fatal to the patient.

The course of the acute form varies greatly. It may rapidly terminate in death by some of the diseases just mentioned, or it may run into the sub-acute or chronic variety, when it is generally *in the*

*end* fatal; or it may yield to treatment, and permanent recovery ensue.

The chronic form most generally commences without any of these febrile symptoms, and often it becomes fully established even before its existence is suspected. It may also commence with subacute febrile symptoms, or succeed to the acute variety. It is incomparably the most frequent form of the disease. Its course is very variable. It has probably been cured, or rather its farther progress has been arrested; but this is not by any means the general result of treatment. The lamentable fact is acknowledged by all, that, very commonly, it is sooner or later fatal. In very many acute cases, it is true, the patient is so much relieved by treatment that he is often confident of having obtained a perfect cure, being so free from all obvious symptoms of the disease. But it requires the experience and knowledge of a physician to discover the latent malady, which, unfortunately, is soon brought to light by the necessary exposure of active life. The organic change proceeds from one portion to another of the kidneys, until they are no longer capable of executing their functions, and death is the consequence. This progress is subject to much inequality, on account, no doubt, of the difference there is in the different cases, in the constitutional procli vity to the disease, in the habits of the patients, in their avoidance of all the exciting causes, and in their ability to follow proper hygienic rules. Where the progress is slow, a patient may live along for a number of years, in an indifferent state of health, with a disposition to œdema and the discharge of albumen in his urine. The first attack of dropsy may generally be removed; so (although with less probability) may the second and third; but finally our treatment fails—the dropsy persists, until the patient is carried off by some secondary complaint,

*Of the Anasarca.*—The first symptom which ordinarily leads us to suspect this form of renal disease, is the occurrence of Anasarca. It may shew itself simply as an œdema of the face and eyelids, or of the hands or feet, and after a few hours or days disappear. Or, as in the acute form, it may be more severe and persistent, invading almost simultaneously a large part of the cutaneous cellular tissue of the body. There may also be effusions into the serous cavities ; or possibly the effusion may be solely into the cavity of the peritoneum. The more acute the disease, the earlier in its course, and the more rapid and extensive is the occurrence of the anasarca.

While anasarca is so common a symptom as to occur in almost every case of this disease, it is not by any means specially characteristic. In some rare cases it does not occur at all, or not until the last few days before death. It is also a symptom of numerous other diseases.

I know of nothing peculiar in the characters of the anasarca from chronic renal disease, that can be depended upon to distinguish it from the anasarca from other causes. Authors almost unanimously describe it as first showing itself in the face or eyelids, while that from disease of the heart as generally first occurs in ths ankles. But this is not always the case ; and the general fact cannot, therefore, be depended upon to aid us in our diagnosis of any particular case. It is said by Rayer, that the œdema of the lower extremities from renal disease does not so readily subside after assuming the recumbent position, as that from heart disease.

In acute dropsy, however, the anasarca seems to have some peculiar characteristics. *First*, in its general and sudden invasion of different parts of the body ; *second*, in a kind of hardness and elasticity of the œdematous parts. They do not so readily pit on pressure as in anasarca from other causes, and when the finger is removed, the impression is much more quickly obliterated.

Anasarca with these characteristics, and accompanied with febrile symptoms, affords strong presumptive evidence of the acute variety of Bright's disease. The diagnosis becomes positive, if, at the same time, the urine is scanty, bloody, and albuminous.

*Of the Urine.*—In acute cases there is a diminution in the quantity of urinary secretion ; occasionally this diminution amounts almost to an entire suppression.

It has generally a smoky, or brownish appearance, from the admixture of blood changed in color by an acid state of the urine.—Sometimes it is reddish ; or the blood may be seen only as a thin layer on the bottom of the vessel, not being so intimately mixed with the urine as to give it a general reddish tinge. Filaments or threads of the fibrin of the blood are occasionally noticed floating in the bottom of the vessel. Deposits of the lithates are frequent. The quantity of urea is not generally diminished in proportion to the amount of urine discharged. The urine is generally acid. Rayer says it is always so. Christison describes it as occasionally neutral or alkaline even. Its specific gravity or density is not materially changed; while, at the same time, according to Christison, there is a material diminution of the daily discharge of the solid ingredients of the urine. In the acute stage also, the urine is always highly coagulable.

The characteristics of the urine vary in several important particulars in the chronic form of the disease. There is rather an increase than a diminution in the daily discharge of the urine; occasionally this increase amounts, during the course of the disease, to a true diabetes-insipidus. Instead of being smoky or reddish, it has a citrine tint, or even becomes pale and transparent. A pale, abundant urine, with general anasarca is looked upon as a condition of disease usually hopeless. Towards the close of the case, as it is drawing to a fatal termination, the urine becomes scanty, often very much so; or, if there be an exacerbation during the disease, the quantity discharged is diminished, and becomes discolored.

Globules of fat are sometimes diffused through the urine, rendering it partially opaque. Deposits of the lithates are rare. There is always a material reduction of the amount of the solids discharged daily with the urine. It may be one-half or three-fourths even. For this reason, its specific gravity is very uniformly low. The urine is generally neutral, or only feebly acid. It is coagulable to a greater or less degree, during the course of the disease, according to the amount of albumen discharged.

The amount of the albumen does not increase as the disease progresses. It is found most uniformly acid in the largest quantity in acute cases, or during an excerbation in the course of a chronic case. It is greatly reduced towards the termination of chronic cases, if not entirely absent; occasionally, also, it cannot be detected during the middle period of such cases. It occurs, besides, in many other diseases, and is not, therefore, an invariable indication of Bright's disease, as has been too commonly supposed. Of what value, then, is it as a symptom? As hemoptysis is valuable as a symptom of Pulmonary phthisis, although not of itself pathognomonic, so is this albuminous discharge from the kidneys valuable as affording a strong probability of this form of renal disease. This probability is rendered almost a certainty, if the discharge is constant and in considerable quantity.

There are two means of detecting albumen, which are very simple, and readily tried. The first is, by heating a portion of the urine up to the boiling point. The coagulation is noticed before the heat reaches this point, and becomes perfect at that heat. A good way of trying it is by decanting some of the urine into a vial, and setting it into some water, which is afterwards boiled.

There are different degrees of coagulation. The urine may be changed almost entirely into a gelatinous mass. A common degree of coagulation is where the precipitate occupies a fourth or half of the fluid. The smallest degree is where a mere haziness is produced.

The second principal test is nitric acid. This is poured slowly into the urine, and when albumen is present, a white precipitate is immediately produced. Either of these tests may fail under particular circumstances. In the first place, if there be a large amount of the phosphates in the urine, as sometimes happens, the test by boiling will cause a precipitate of these salts which might be confounded with albumen. In this case, the nitric acid would redissolve the precipitate, and the urine would become clear. And again, if the urine contained a large portion of the lithates, and nitric acid were the test used, it would be apt to cause a deposit of lithic acid, which would be redissolved by heating. Thus it is seen, that these tests of albumen are also tests of each other. Where both cause a deposit in different specimens of the same urine, that deposit is albuminous.

It is said there are some cases of alkaline urine holding albumen in solution, where both these tests fail, if used separately. In such an instance the nitric acid ought to be added just sufficiently to render the urine acid, and then apply heat, and the precipitate will be formed. Other tests have been proposed, but these are the only ones that will be needed in the immense majority of cases.

I have already referred to the reduction of the average daily discharge of the solid constituents of the urine as being characteristic of this disease. Its importance demands for it some farther notice. The amount of the solids discharged average about two and a half ounces daily in healthy persons of the ordinary physical developement. In this disease, the diminution is always material, and may be reduced, according to Christison, to a third of an ounce, or even to one drachm in twenty-four hours. This diminution seems to affect all the principles of the urine indiscriminately. It is difficult to ascertain the exact amount of the solids discharged, although often desirable to do so. Were the amount of the urinary secretion always the same, its specific gravity would at once determine this point.—But this is very variable, and its density may be high if the quantity of urine be small, although the daily discharge of the solids be much reduced.

To determine the amount of the solids discharged, is therefore a labor requiring much caution and considerable delicacy of manipulation. Those who have gone through with the work, have discovered the important law that in this disease the density of the urine declines as the disease advances. So uniformly is this the case, that if a patient have all the general appearances of this disease, with a low density of the urine, we would decide that he had it, although no albumen could be detected in his urine. And again, if a patient have an albuminous urine which has not a low density, and has none of the general symptoms of the disease, we would not be justified in declaring that he had the disease.

*Of the Blood.*—While these changes are going on in the urine, the blood also becomes altered. The first change noticed, is a great and rapid diminution of the red globules. The patient becomes leucophlegmatic; his complexion has a waxen appearance, which is very characteristic of the disease when of some standing, and is often of itself sufficient, at first sight, to determine the mind of the physician as to his patient's malady. The red globules become reduced from the healthy standard of 134 parts in 1000, to 100, 95, 72, and even lower. There is no disease except hemorrhage, which so impoverishes the coloring particles of the blood.

The fibrin of the blood is increased in the acute form, as shewn by a strong buffy coat on the blood drawn. The serum is then often lactescent from the intermixture of fat globules.

A definite but inverse relation exists between the density of the serum and the coagulability of the urine. In acute attacks, when there is such an increase of the albumen in the urine, the density of the serum becomes correspondingly low ; while in the middle stages of the chronic form of the disease, when there is but little albumen discharged from the kidneys, the serum of the blood has its natural specific gravity.

Another important characteristic of the blood is the accumulation of urea in it. This substance is found in the system, and excreted by the kidneys. But in acute cases, and in the latter stages of chronic cases, or whenever the daily discharge of the urine has been diminished to about one-third of its healthy average, it can be detected in the blood. This urea is supposed to act as a poison upon the nervous system, and undoubtedly occasionally causes coma and those brain symptoms which so often are the precursors of a fatal termination.

An increase of the fibrin, and a diminished density of the serum, whenever inflammatory symptoms exist—a diminution of the hematosin of the blood, the extent of which affords perhaps the best criterion of any one symptom of the stage of the disease—an increase of the urea in the blood, whenever there is a marked diminution of the secretion of the urine, are the main alterations of the blood, so far as at present known.

*Of the Secondary Diseases.*—These constitute some of the most important points in the history of Bright's disease. They form a numerous class of diseases which require especial attention in the treatment of every case : they are occasionally latent— more generally are easy of detection, and difficult to treat successfully, and consequently are frequently fatal in themselves. They are in fact the chief sources of danger through the middle stages of the disease; and patients often die of them without the primary disease of the kidneys being suspected.

There are two classes of them : those that are distinctly inflammatory, and those which partake more of functional disorder. Both varieties may complicate the acute and chronic forms of Bright's Disease.

Under the first class may be enumerated inflammations of the different serous membranes of the body, a form of complication more frequent than any other. They are pleurisy, peritonitis, pericarditis, and meningitis. The only other inflammation frequently seen is pneumonia of hybrid form, partaking of the character of congestion as well as of inflammation. This is frequently seen in the anasarca of children after scarlatina.

The secondary diseases of a chronic nature consist mostly of disorders of the digestive mucous membrane. These are dyspepsia, with great irritability of the stomach, and vomiting; and diarrhœa, dysentery connected with chronic inflammation, and ulceration of the intestinal mucous membrane. Under the head of 'Causes,' I referred briefly to the complication of diseases of the heart and of the liver. The existence of any of these complications often greatly embarrasses us in our treatment, and adds in an especial manner to the miseries and discomforts of our patient.

Chronic rheumatism seems so frequently connected with the advanced stages of this disease, according to Christison's experience, that he recommends that the condition of the urine be inquired into in all cases of obstinate rheumatic affections.

I have purposely deferred the consideration of the brain symptoms which so frequently complicate this disease, until an account had been given of the symptoms, and more particularly of the alterations the blood undergoes as the disease proceeds to a fatal termination. By some, these brain symptoms are given under the head of symptoms—others classify them among the secondary diseases When they arise from congestion, or from a meningitis, or from an apoplectic effusion, whether sanguineous or serous, they undoubtedly belong to the class of secondary diseases. But in the majority of cases, no such lesions can be found, although the symptoms of a cerebral lesion have been well marked. The autopsy shews merely an unusual paleness of the brain, and a want of blood in the cerebral vessels (Christison). The symptoms then arise as a direct consequence of changes which the blood has undergone from the progress of the disease, and ought really to be enumerated under the head of symptoms.

I have preferred, however, to bring them all together at this point in the history of the disease, and to classify them under the heads of Symptoms from cerebral lesion, and Symptoms from the altered condition of the blood in the last stages.

These symptoms are so common when it ends in death, as to be considered, by some, the natural mode of termination of the disease. Of seventy cases in which Dr. Bright was able to trace the circumstances connected with the conclusion of life, no less than thirty died of well-marked symptoms of cerebral derangements, noted under the titles of 'Apoplexy,' 'Coma,' 'Convulsion,' and 'Epilepsy.' Besides these, thirty-eight others died suddenly, probably from some sudden commotion or change in the nervous system.

These cerebral symptoms may come on suddenly by an attack of convulsions; or, as is more generally the case, their approach is first indicated by an "insidious drowsiness," which is soon followed by stupor, then coma, and death with or without convulsions. Sometimes an attack of apoplexy, with hemiplegia, and a more or less entire loss of consciousness precedes death for a few days; or epileptic convulsions closes the scene. In chronic cases, these head symptoms generally last a week before proving fatal. When they occur in the acute form of the disease, they are more rapidly fatal, unless, as may frequently be done, they are arrested by treatment.

Grisolle notices some cases of death by brain symptoms, where the disease of the kidneys was latent; where the only symptoms noticed were a loss of flesh and strength, without anasarca or other symptoms which would direct attention to the kidneys as the suffering organs. "We see these persons," he says, "taken suddenly "with cerebral symptoms, or some of the acute affections of the "chest, to which they rapidly succumb; and at the autopsy we find "the characteristic change in the kidneys, which explains all the "symptoms observed during life. The facts are not rare; we have "often observed them, and they ought to cause the physician when "he is searching for the organic cause of a condition of bad health "and emaciation, to interrogate the urinary secretion, as well as "the other functions.'.

*Treatment.*—Under this head I purpose hurriedly to notice the principal remedial indications, without being at all minute. I would refer the reader, for farther particulars, to a paper by Christison, on the "Granular Disease of the Kidney," in the Library of Practical Medicine, and to the Chapter on Renal Dropsy, by Watson, in his Practice of Medicine. They are the only monographs on this subject at present generally accessible to the practitioners of this country.

There is no discrepancy, I believe, as to the course to be pursued in the treatment of the acute form of the disease, whether it be the result of common causes or of Scarlet Fever. It is to be strictly antiphlogistic ; governed, of course, by the acuteness of the attack, the amount of the fever, and the rapidity and hardness of the pulse. We are to treat it as an inflammation of the kidneys. Venesection is almost always demanded, except in young children, when free leeching will often answer. The bleeding, whether by the lancet or leeches, may be required to be repeated two or three times. Cups or leeches to the loins may sometimes be used with great advantage. The bowels are to be freely acted upon ; and a solution of antimony, or of antimony and nitre ought to be given at short intervals. The saline diuretics are useful after the force of the circulation has been removed. Digitalis is highly recommended.

Perspiration is to be promoted by the usual means.

Various symptoms may arise which will require some modification of the course here marked out. The experienced physician will not fail to make such modifications, especially as the case approaches towards a cure, or runs into a chronic form. The first indications of any of the secondary diseases, ought to be constantly watched for, and promptly met.

When the disease becomes, or is from the first, chronic, the treatment is extremely difficult ; and although often highly beneficial to the patient—as far as relieving him from many pains and miseries, and often thereby prolonging his life—it is yet almost totally inefficacious in producing a radical cure. The remedy to effect this great object is still to be found.

The dropsy is the one symptom from which it is our patient's great anxiety to be relieved ; and it is to this that our principal exertions generally tend. If there is much inflammatory action, we must first reduce it by the application of cups to the loins, a purgative, and the use of antimony or of Dov. powder as a diaphorettic. Then, for the removal of the dropsy, we have various agents we may administer, but all requiring more or less caution, and all being more or less uncertain and variable in their action in different cases.

1st. *Of Purgatives.*—They are very valuable when borne, but the difficulty with them is, they are apt to produce a distressing diarrhœa or dysentery. Great caution is required on this account. If, in any case, there is a tendency to diarrhœa, it would be better to abstain from them altogether, while such tendency continued. Case 1, in the June No. of this Journal, is an instructive one in this particular. The purgatives most recommended by Dr. Bright are the Elaterium and Jalap. The saline purgatives are useful in reducing the anasarca ; the old-fashioned combination of Jalap and Cream of Taatar is one of the best purgatives for this purpose. Gamboge, and Croton Oil, sometimes answer a very good purpose. Without an exception almost, do authors condemn the use of mercury. It has long been thought capable of causing albuminous urine. It is positive that it affects the system in this disease with great facility and severity ; and it is said to be beneficial neither to the action of the skin nor to that of the kidney. Nevertheless, there may be circumstances, more especially in acute cases, where it would do good. Dr. Osborne says he has found it beneficial in obviating effusion in affection of the head. In some cases also where other cathartics fail to act, or cannot be taken on account of great irritability of the stomach, it may be given with advantage. Of such, or other special indications for its administration, the physician must judge in each particular case.

2d. *Of Diuretics.*—The use of this class of remedies is condemned by Osborne, Bright and Rayer, as being likely to add to the already existing irritation of the kidneys. Christison alone is inclined to recommend them in certain cases where dropsy prevails, or coma is threatened, with great decrease of the urine. In chronic cases with an increase of the natural secretion of urine, their use would hardly seem to be demanded as mere diuretics ; but as a tonic to the kidneys, to effect a change in their action, the mineral acids, the Tr. Ferri Chloridi, or Tr. Cantharides, Copaiba, the Uva-ursi or Buchu, with or without soda, might be beneficially prescribed. In cases of diminution of urine, with a tendency to brain symptoms, or with general and large anasarca, I would not hesitate to prescribe them, as Christison advises, especially if in moderate quantities they produce an amelioration of the symptoms for which they were given. The diuretics to be given in such cases, are the Bitartrate of Potass, in an infusion of Juniper-berries, the Sp'ts of Nitre and the Digitalis. They occasionally fail in relieving the patient in sub-acute cases, or during exacerbations, when a general bleeding has been followed by subsidence of all the swelling, and a general improvement in all other respects.

3d. *Of Diaphoretics.*—As the general opinion is that the dropsical effusion is produced, secondarily, by disordered action of the skin, so all who have seen the most of the disease insist upon that course of measures which tends directly to restore the functions of the skin to a healthy and perspirable state. One writer (Osborne) even says that he has "never failed in removing this kind of general dropsy, whenever the entire surface of the body was restored to a perspiring state;" and he accordingly strongly recommends the diaphoretic treatment. No other one seems to have succeeded so well with diaphoretics, although often successful with them in dispersing the anasarca.

The diaphoretic treatment consists in a strict confinement to bed, and the use of various external means to promote sweating. These are partial and general warm baths, the vapor bath, the hot-air bath, warm applications, as poultices, kept constantly to the abdomen and loins, and frictions to the limbs, with some stimulating liniament. These external applications are assisted by the administration of diaphoretic medicines, the principal of which are the Dov. Powders, small doses of Antimony, Spirits of Nitre, and the Spirit of Mindererus. When there is coldness of the extremities and surface, preparations of Guiaci and Camphor may be useful. So much for the treatment of the disease, as usually seen by the physician, complicated with anasarca.

The first class of the secondary diseases require the usual treatment of phlegmasiæ, the activity and extent of our depletions being modified, of course, by the stage of the renal disease.

The second class are usually particularly obstinate. The stomach affections require bitters and antacids — sometimes stimulants. Hydrocyanic acid, creosote, or opium, will occasionally subdue the chronic vomiting.

The diarrhœa and dysentery are to be treated by opiates and astringents, with occasional laxatives. Anodyne injections will often be found particularly serviceable. Again, neither opium, nor astringents, nor enemas, will give more than temporary relief; and after a few days or a few weeks, our patient will die of the most distressing of all complications.

For the rheumatism, no one method of treatment is even generally successful, (Christison).

The chief remedies for the brain symptoms are general depletion, if there is much activity of the circulation: local depletion, by cups or leeches, are generally useful in relieving the symptoms for a time: brisk purgatives, and the more active diuretics: applications of ice or of ice-water to the head, may assist us, as also the drawing of a blister on the neck.

We have no direct curative agents for the disease of the kidneys. We have no means that we know of, either of procuring absorption of the deposit, or of changing the morbid action of which that deposit is the result. Where the disease is suspected, or has commenced, we have only the general hygienic means that we oppose to phthisis, or to any other chronic incurable malady. The utmost circumspection is required to avoid all exciting causes: every means must be adopted to keep the system up to a proper standard of health, by diet, exercise, frequent bathing, daily friction of the skin by the hair-gloves, or the common flesh-brush, or even by coarse towels; the constant protection of the surface by flannels; and, finally, by seeking a climate free from the sudden changes of our northern latitudes.

